# Impact on Air Quality of the COVID-19 Lockdown in the Urban Area of Palermo (Italy)

**DOI:** 10.3390/ijerph17207375

**Published:** 2020-10-09

**Authors:** Marcello Vultaggio, Daniela Varrica, Maria Grazia Alaimo

**Affiliations:** 1 Risorse Ambiente Palermo (RAP), Piazzetta B. Cairoli, 90123 Palermo, Italy; marcello.vultaggio@rapspa.it; 2 Dipartimento di Scienze della Terra e del Mare (DiSTeM), via Archirafi 22, 90123 Palermo, Italy; mariagrazia.alaimo@unipa.it

**Keywords:** COVID-19, air quality, lockdown, CO–NO_2_–O_3_–PM_10_, urban environment

## Abstract

At the end of 2019, the first cases of coronavirus disease (COVID-19) were reported in Wuhan, China. Thereafter, the number of infected people increased rapidly, and the outbreak turned into a national crisis, with infected individuals all over the country. The COVID-19 global pandemic produced extreme changes in human behavior that affected air quality. Human mobility and production activities decreased significantly, and many regions recorded significant reductions in air pollution. The goal of our investigation was to evaluate the impact of the COVID-19 lockdown on the concentrations of the main air pollutants in the urban area of Palermo (Italy). In this study, the trends in the average concentrations of CO, NO_2_, O_3_, and PM_10_ in the air from 1 January 2020 to 31 July 2020 were compared with the corresponding average values detected at the same monitoring stations in Palermo during the previous five years (2015–2019). During the lockdown period (10 March–30 April), we observed a decrease in the concentrations of CO, NO_2_, and particulate matter (PM)_10_, calculated to be about 51%, 50%, and 45%, respectively. This confirms that air pollution in an urban area is predominantly linked to vehicular traffic.

## 1. Introduction

The new severe acute respiratory syndrome-coronavirus (SARS-CoV2) has had an unprecedented impact around the world. The spread of the 2019 coronavirus disease (COVID-19), initially identified in Wuhan in China, led to over one million cases worldwide in the first four months [1]. The virus has affected almost all countries on the planet (235 in total), causing so far over 34 million confirmed cases and about 1,030,000 deaths [2]. This has resulted in lockdown in many nations. The COVID-19 pandemic has substantially affected the human society, including healthcare, economic structures, and social relationships [3].

In Italy, between the months of February and March, a series of national and regional legislative measures were issued to stop the spread of COVID-19, which radically changed the daily habits and lifestyles of citizens. The lockdown and the related implemented measures led to a sudden drop in economic activities, including a fall in road transport in many cities and a drastic reduction in the movement of citizens through the adoption of smart-working practices wherever possible.

In Italy, the Decree of the President of the Council of Ministers (DPCM) of 9 March 2020 [4], also known as the “I stay at home” Decree, extended the measures on confinement and restriction on travel to the whole country and prohibited any form of gathering of people in public places or locations open to the public, also suspending sporting and cultural events. The DPCM of 21 March 2020 [5], known as “Italy’s block”, blocked non-essential activities and imposed a ban on moving to different municipalities. In Italy, lockdown and travel restrictions were maintained until 3 May 2020.

Is lockdown a factor influencing air pollution? 

Reducing air pollution could be considered part of an integrated approach for the protection of human health and the prevention of epidemic spread. The role of air pollution in contributing to the high levels of SARS-CoV-2 lethality in northern Italy has been hypothesized [6,7].

Exposure to air pollution is an important and persistent risk factor for death by different causes and for a high incidence of respiratory diseases [8,9]. Deaths associated with air pollution include aggravated asthma, bronchitis, emphysema, lung and heart disease, and respiratory allergies [8]. Among the atmospheric pollutants, the focus is mainly on nitrogen dioxide (NO_2_), particulate matter (PM_2.5_ and PM_10_), and ozone (O_3_), which are frequently found at high concentrations in urban areas.

Various studies have reported a direct relationship between the spreading and contagion capacity of some viruses and the atmospheric levels and mobility of atmospheric pollutants [10,11]. The results provided by Zhu et al. [12] indicated that a significant relationship exists between air pollution and COVID-19 infection, which could partially explain the effect of the national block and provide implications for the control and prevention of this new disease.

A decline in anthropogenic air pollution has been observed in countries that have responded to the COVID-19 pandemic with a total closure of all activities. Satellite data recorded by the NASA Earth Observatory showed that NO_2_ concentrations in eastern and central China from the beginning of 2020 were 10–30% lower than those in comparable periods in 2019 [13]. In particular, in Hubei province (China), strict social distancing measures that affected the main economic activities of the region were implemented from December 2019; power plants and industrial structures stopped production, and the use of vehicles decreased considerably [14]. This led to a drastic reduction in the levels of NO_2_ and fine atmospheric particulate matter [15,16]. The European Environment Agency found a similar decrease in air pollution in European cities [17]. Air pollution drastically reduced as major industries and other regular businesses came to a halt, leading to a sharp reduction in NO_2_ concentrations in countries such as France, Germany, Italy, and Spain [14,18,19]. From 16 to 22 March 2020, Bergamo (Italy) and Barcelona (Spain) showed decreases in NO_2_ of 47% and 55% compared to the same dates in 2019, respectively. The impact of the COVID-19 pandemic on air pollution measured by the United States using the federal air monitoring network showed strong reductions in fine particulate matter (PM_2.5_) and NO_2_, corresponding to reduced traffic and mandatory company closures [20].

In our investigation, we explored the impact of COVID-19-relatd restrictions on the main air pollutant concentrations, CO, NO_2_, O_3_, and PM_10_, measured in the city of Palermo (Italy) using the municipal air-monitoring network. We used statistical and quantitative analyses of the relationships between air pollution, human mobility, and travel restrictions to infer the effects on air quality.

The objectives of this study were: (1) to compare the concentrations of the main atmospheric pollutants determined in the city of Palermo (Italy) during the pre-lockdown, lockdown, and post-lockdown periods; (2) compare the collected data with those of the same time window in the previous five years (2015–2019); and (3) evaluate the usefulness of the block as an alternative strategy for reducing the level of atmospheric pollution in the city of Palermo.

## 2. Materials and Methods

### Description of the Area and Sampling Sites

With about 680,000 inhabitants, Palermo is the largest urban area of Sicily, and its metropolitan area is populated by more than one million people. The city is situated on the north-western coast of the island, facing the Tyrrhenian Sea on the northeast and surrounded by mountains (Monti di Palermo) reaching 500–1000 m above sea level. The climate is typically Mediterranean, with warm dry summers and moderately rainy winters. The prevailing wind directions are from the east and west. The city of Palermo is periodically affected by frequent warm winds coming from the south-east (Sirocco winds) and the southwest (Libeccio winds), carrying dust raised from the Sahara Desert region throughout the Mediterranean basin, which considerably influences the concentration of PM_10_. Potential local pollutants are limited to emissions from traffic, domestic heating, and small manufacturing industries.

We decided to compare the data collected from monitoring stations during the period from 1 January 2020 to 31 July 2020 with the averages for the same period calculated for the five-year period of 2015–2019. 

The CO, NO_2_, and O_3_ values were recorded hourly, whereas the PM_10_ values were recorded daily. Each hourly or daily value for the five-year period is the result of the average calculated from all the values recorded in the same hour or day in the considered five years. From the data of the five-year period, the values due to abnormal and/or exceptional weather conditions and not attributable to anthropogenic emissions were deleted. 

The comparison was performed using data on pollutants collected from monitoring stations, shown in Table 1. In total, 213 days were analyzed for CO, NO_2_, O_3_, and PM_10_ in urban stations. The selected urban stations are close to roads with different densities of vehicles, urban buses, and extra-urban buses.

Gas analyses were performed using the municipal air quality monitoring network managed by Risorse Ambiente Palermo (RAP). Ambient CO concentration measurements were recorded according to European standard EN 14626; NO_2_ concentration was analyzed according to European standard EN 14211 [21]. Ambient O_3_ concentration measurements were recorded according to European standard EN 14625 via ultraviolet photometry. PM_10_ was sampled with a beta gauge analyzer (Environnement S.A. MP101M, ENVEA, Italy), certified as equivalent to the reference method in accordance with EN12341 [22] (stations: Di Blasi and Giulio Cesare) and another beta gauge analyzer (OPSIS SM200, OPSIS, Sweden) for the Castelnuovo and Indipendenza stations. 

## 3. Results and Discussion

### 3.1. Gaseous Air Pollutants, CO, NO_2_, and O_3_

The average concentrations of the gaseous air pollutants carbon monoxide (CO) and nitrogen dioxide (NO_2_) were used to verify the impact on pollution of the closure of all activities in a city.

**Figure 1:**
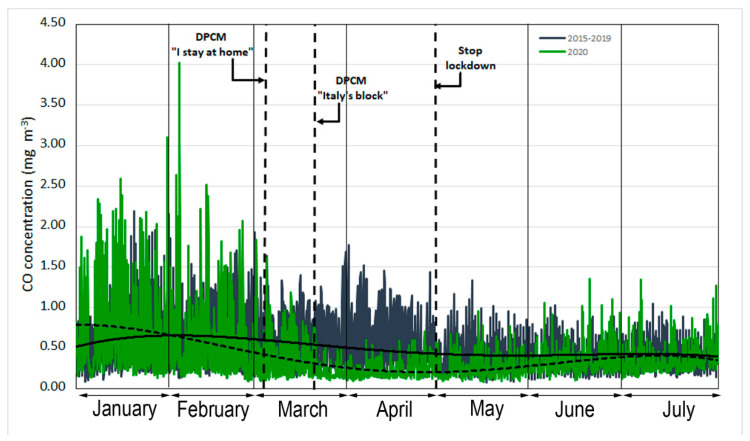
Time series of hourly CO (mg m^−3^) concentrations measured from 1 January to 31 July. The dotted and solid regression lines indicate the 2020 and 2015–2019 trends, respectively.

The main sources of CO emissions are linked to incomplete combustion processes of natural gas, diesel, or gasoline from engines and household heating [23]. In recent years, the levels of CO in Palermo city were below the limit of 10 mg m^−3^ (maximum daily eight-hour mean) fixed by Directive 2008/50/EC [24]. Figure 1 shows the temporal trend in the hourly average concentration of carbon monoxide in the atmosphere, detected for the period from 1 January 2020 to 31 July 2020 for the urban stations compared to the trend of the hourly average concentration resulting from the processing of the data of the five-year period of 2015–2019 for the same months. 

The trend for the hourly concentrations in the city of Palermo showed a drop in the week preceding the Prime Minister’s decree of 9 March 2020. Following the detection of COVID-19 cases since 20 February, the closure of all schools and grades had already been implemented. 

From 10 March (DPCM 9 March 2020 [4], “*I stay at home*”) and even more evident from 22 March (DPCM 21 March 2020 [5] “*Italy’s block*”), which defined the total lockdown, CO concentrations were lower than the corresponding values of the five-year (2015–2019) period, confirming that the main source of CO was heavy vehicular traffic. After the end of the lockdown, we observed that CO concentrations tended to slowly increase until they reached very similar values to those of the month of July of the 2015−2019 period.

**Figure 2:**
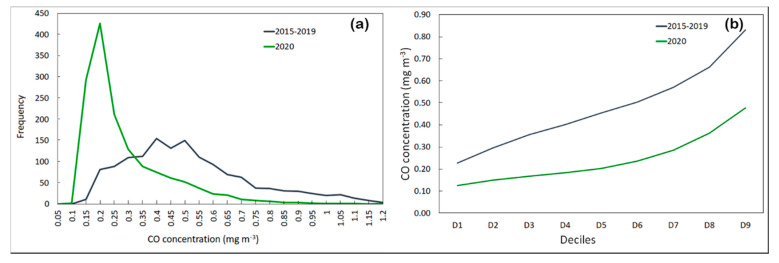
(**a**) Frequency distribution and (**b**) deciles of two temporal trends, from 1 March to 30 April, of CO concentrations (mg m^−3^).

In Figure 2a, we report the frequency distribution of two temporal trends, where the sharp reduction observed during the lockdown period is more evident. Figure 2b plots the deciles calculated for the two trends, showing that D5 was 0.2 mg m^−3^ for the 2020 distribution and 0.47 mg m^−3^ for the 2015−2019 distribution, representing a reduction of −56%.

**Figure 3:**
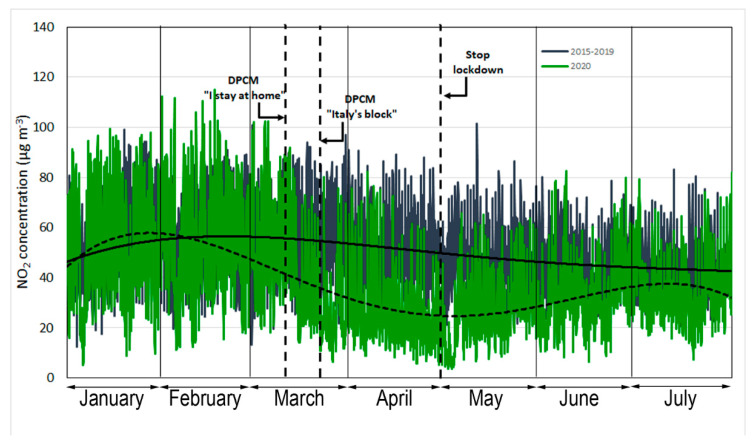
Time series of hourly NO_2_ (µg·m^−3^) concentrations measured from 1 January to 31 July. The dotted and solid regression lines indicate the 2020 and 2015-2019 trends, respectively.

A similar trend was observed for nitrogen dioxide (Figure 3). The average NO_2_ concentrations at the urban stations did not exceed the legal limit of 200 µg·m^−3^ (in one hour) but exceeded the annual mean of 40 µg·m^−3^, imposed by Directive 2008/50/EC [24] in the 2015–2019 period. 

From 1 January to 10 March 2020, the trend in the hourly concentration of NO_2_ can be superimposed on that observed in the prior five-years. Since the DPCM of 9 March 2020, a drastic decrease in the hourly concentration of NO_2_ was observed. Nitrogen dioxide in the atmosphere is a highly reactive pollutant emitted from burning fossil fuels (diesel, gasoline, and coal) [25,26]. This pollutant is considered a good indicator of air pollution in urban areas, as its concentration is heavily influenced by transport and mobility of citizens. At the end of the lockdown, some economic activities did not resume, schools and universities remained closed, and smart working was maintained. This reopening period coincided with the partial emptying of the city for the summer holidays. The density of motor vehicles decreased because of the use of bicycles and scooters.

**Figure 4:**
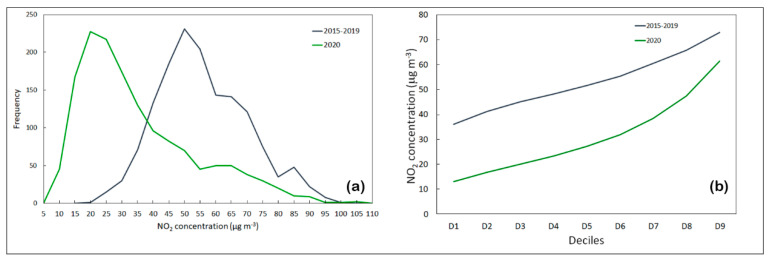
(**a**) Frequency distribution and (**b**) deciles of two temporal trends of NO_2_ concentrations (µg·m^−3^) from 1 March to 30 April.

Figure 4 shows the frequency distribution and deciles of two temporal trends. The trend in NO_2_ concentration observed is similar for the two periods, but with a strong concentration reduction in 2020. The concentrations decreased significantly during the lockdown due to the stoppage of transport and low mobility [27,28].

Table 2 reports the average values for CO and NO_2_ pollutants at the individual monitoring stations. The measured values showed that the link between improved air quality and traffic density was more visible in the DB and GC monitoring stations, with variations during the lockdown between −58% and −43% for CO and −37% and −54 % for NO_2_.

The statistical descriptions for CO and NO_2_ considering the period 1 March–30 April, the period after the first DPCM (10 March–30 April), and that after the second DPCM (22 March−30 April) are provided in Table 3. The calculated percentage changes show a strong decrease in carbon monoxide and nitrogen dioxide between the periods considered. The two-tailed paired *t*-test showed that the observed differences among the three groups were statistically significant at *p* < 0.05.

**Figure 5:**
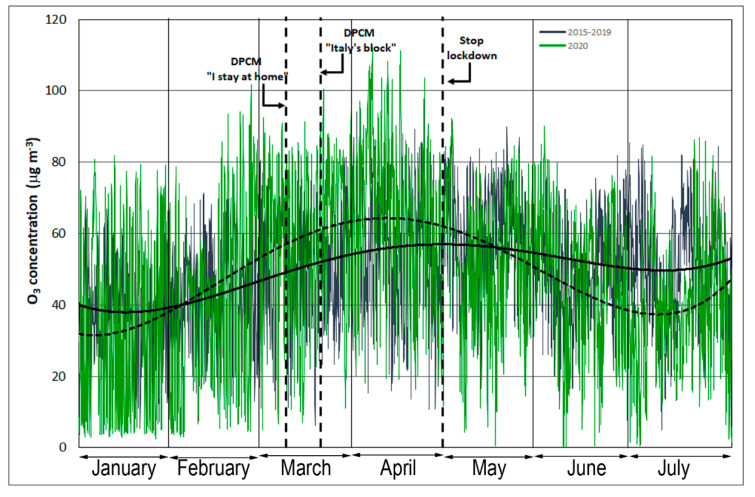
Time series of hourly O_3_ (µg·m^−3^) concentrations measured from 1 January to 31 July at the CS monitoring station. The dotted and solid regression lines indicate the 2020 and 2015-2019 trends, respectively.

**Figure 6:**
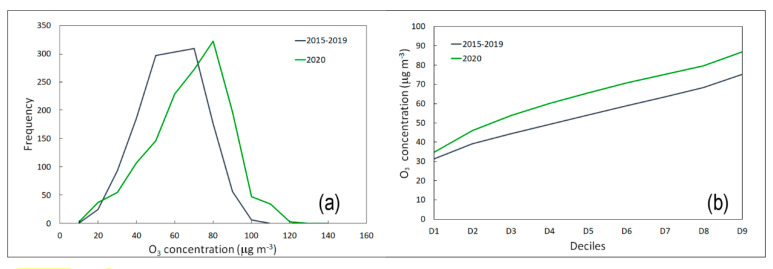
(**a**) Frequency distribution and (**b**) deciles of two temporal trends from 1 March to 30 April of O_3_ concentrations (µg·m^−3^) from 1 March to 30 April.

Ozone levels have increased as a result of human activities. Photochemical processes influenced by anthropogenic emissions of ozone precursors (NO_2_ and volatile organic compounds (VOCs)) have caused the current tropospheric ozone levels to be substantially higher compared to the natural background levels [29]. The critical aspect of ozone is related to its complex photochemistry, as the rates of ozone formation and accumulation are non-linear functions of the mixture of VOCs and NO_x_ in the atmosphere [30]. Under certain conditions, NO_x_ reduction can lead to higher ambient ozone levels [31]. Motor vehicles are the main source of NO_x_ emissions in an urban area; the increase in ozone production has been linked to the reduction in vehicular traffic [32,33]. The increase in ozone concentration that occurred during the lockdown is highlighted in Figure 5 and Figure 6.

The temporal analysis of the O_3_ level showed an opposite behavior during the block (63 µg·m*^−3^*) compared to the average values of NO_2_ (21 µg·m*^−^*^3^) at the same monitoring station. An increase in O_3_ is usual during spring and summer due to the higher solar radiation (in terms of intensity and daily duration), which promotes the photolysis of NO_2_ [34,35]. The increase in ozone concentrations registered in Palermo during the lockdown is linked to two factors: the decrease in NO_2_ and the increased solar radiation [36]. 

### 3.2. Particulate Matter PM_10_

Particulate matter in urban areas is mainly composed of mineral dust, metals, metalloids, sea salts, ammonium nitrate and sulfate, organic compounds, and elemental carbon [37]. Anthropogenic airborne particulate matter comes from a variety of sources, including traffic, industry, commerce, and domestic heating [23].

**Figure 7:**
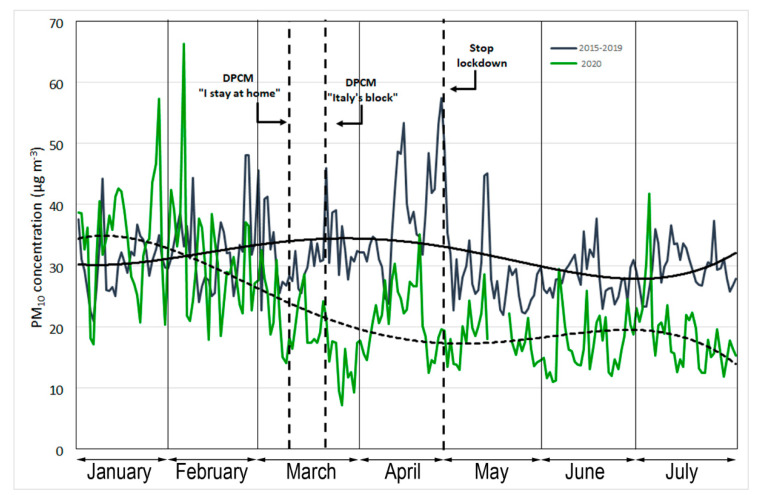
The daily values of ambient concentrations of PM_10_ (µg·m^−3^) measured from 1 January to 31 July. The dotted and solid regression lines indicate the 2020 and 2015-2019 trends, respectively.

**Figure 8:**
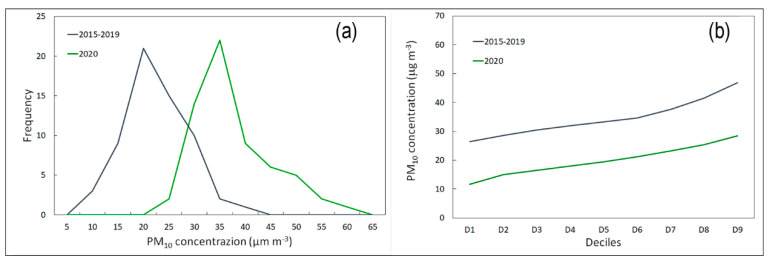
(**a**) Frequency distribution and (**b**) deciles of two temporal trends of PM_10_ concentrations (µg·m^−3^) from 1 March to 30 April.

The daily variation in PM_10_ from 1 January to 31 July for the average values of 2015–2019 and for 2020 is shown in Figure 7. The mean concentration of particulate matter from 1 March to 30 April was higher during 2015–2019 (35 µg·m^−3^) than in 2020 (20 µg·m^−3^), representing a change of −43%. The effect of movement restrictions imposed by the authorities from 9 March and subsequently from 21 March marked a further overall decrease in PM_10_ of 45–48% compared to the same period in 2015–2019. The average values measured from 9 March to 30 April were 36 µg·m^−3^ (2015–2019) and 20 µg·m^−3^ (2020); from 21 March to 30 April, the mean measured concentrations were 37 µg·m^−3^ (2015–2019) and 19 µg·m^−3^ (2020). For the period following the end of the lockdown (1 May to 31 July), the average measured values were 29 µg·m^−3^ (2015–2019) and 18 µg·m^−3^ (2020), which is a change of −60%. Figure 8 shows the significant difference between the two frequency distributions. In particular, the median values report concentrations of 35 µg·m^−3^ (2015−2019) and 19.4 µg·m^−3^ (2020). 

The 24 h average daily values of ambient concentrations of PM_10_, measured at each of the four sampling sites during the study period, are reported in Table 4. The GC, IND, and DB sites showed higher percentage changes of −43%, −44%, and −53%, respectively, between 1 March and 30 April.

These results can be attributed to the reduction in vehicular traffic as a consequence of the closure of all schools and universities and the decrease in workers’ movements. This hypothesis is confirmed by several previous studies conducted in the same area, in which the background value for PM_10_ was estimated to be around 23 µg·m^−3^ on the basis of a linear correlation between PM_10_ and CO and NO_2_ [38,39]. Thus, the constant presence of a fraction of PM_10_, not strictly related to vehicle emissions, could result from soil and road dust particle resuspension. Dongarra et al. [39] confirmed that about 50% of particulate matter is produced by road traffic.

The reported data highlight the changes in air quality during the lockdown period. Strict alternative measures could be envisaged, such as short-term blocks, for pollution reduction in urban air. Table 5 shows that the decreases in CO, NO_2_, and PM_10_ concentrations can be evaluated starting from the first six days of block. A statistical evaluation was conducted, which highlighted percentage changes of concentrations between 31% and 66% for CO, between 30% and 52% for NO_2_, and between 24% and 59% for PM_10_. The *t*-test confirmed that the variations observed CO, NO_2_, and PM_10_ between the lockdown period and the previous five years were statistically significant (2015–2019; *p* < 0.05).

### 3.3. Pre-Lockdown

To verify the findings of the analysis of this study and the reliability and representativeness of the 2015–2019 database, a statistical analysis was also conducted to check the percentage changes for each pollutant from 1 January to 29 February 2020. During this period, the city of Palermo was not affected by any restrictions related to the COVID-19 pandemic. The results obtained showed that in the period considered, the differences between the average and the median between the two data series were practically negligible, and the variations were all less than 6% in absolute value for the arithmetic means and 10% for the medians, as shown in Table 6. The data obtained confirmed, despite the many variables involved, the reliability and representativeness of the 2015–2019 database used for the comparison.

## 4. Conclusions

Air pollution studies should be considered as part of an integrated approach for sustainable development and the protection of human health. The national lockdown during the COVID-19 pandemic provided an opportunity to work on improving air quality. The results of this study demonstrate that anthropogenic activities strongly influence air quality. The lockdown period produced a sharp reduction in all pollutants that are closely linked to vehicular traffic. In the urban area of Palermo (Italy) from 10 March to 30 April, the concentrations of CO, NO_2_, and PM_10_ decreased to about 51%, 50%, and 45%, respectively. The period of forced block produced decreases in the concentrations of CO, NO_2_, and PM_10_ from the first six days of lockdown. The lack of a decrease of tropospheric ozone during the block due to non-linear chemical effects showed that these reductions will remain challenging, even with effective policies to reduce primary pollutants.

The findings reported here are a useful indication for competent authorities to rethink existing regulatory plans and provide assurance for the implementation of rigorous alternative measures such as short-term blocks to produce a real improvement in air quality.

## Figures and Tables

**Table 1:** Sampling site location for CO, NO_2_, O_3_, and particulate matter (PM)_10._

Pollutant	Monitoring Station		
CO	Di Blasi	DB	Urban (Hight density traffic)
	Giulio Cesare	GC	Urban (Heavy density traffic)
	Indipendenza	IND	Urban (Lower traffic flow)
NO_2_	Castelnuovo	CS	Urban (Heavy density traffic)
	Di Blasi	DB	Urban (Hight density traffic)
	Giulio Cesare	GC	Urban (Heavy density traffic)
O_3_	Castelnuovo	CS	Urban (Heavy density traffic)
PM_10_	Castelnuovo	CS	Urban (Heavy density traffic)
	Di Blasi	DB	Urban (Hight density traffic)
	Giulio Cesare	GC	Urban (Heavy density traffic)
	Indipendenza	IND	Urban (Lower traffic flow)

**Table 2:** Average concentrations of CO and NO_2_ at individual monitoring stations.

		2015–2019				2020				% Change				*t*-Test			
		IND	GC	CS	DB	IND	GC	CS	DB	IND	GC	CS	DB	IND	GC	CS	DB
**CO**	01/01–/02/29	0.49	0.76		0.64	0.48	0.81		0.70	−2	7		9	*0.5*		−2.8	−2.9
	03/01–04/30	0.41	0.63		0.49	0.24	0.26		0.28	−41	−58		−43	17.5		43.6	18.9
	05/01–07/31	0.33	0.51		0.40	0.26	0.32		0.39	−21	−38		−3	9.9		36.0	*1.7*
**NO_2_**	01/01–/02/29		59	50	52		58	41	65		−1	−19	25		*0.7*	13.6	−15.8
	03/01–04/30		56	46	58		29	21	36		−48	−54	−37		42.1	37.2	30.2
	05/01–07/31		45	39	53		31	24	40		−31	−38	−23		33.3	36.2	25.7
**O_3_**	01/01–/02/29			40				39				−4				*1.8*	
	03/01–04/30			54				63				18				−14.9	
	05/01–07/31			53				53				−1				15.1	

Data are expressed in mg·m^−3^ for CO and µg·m^−3^ for NO_2_ and O_3_). Parametric *t*-test at *p* < 0.05. The italics indicates a not significant *t* test.

**Table 3:** Main statistical parameters of CO and NO_2_; results of the parametric *t*-test at *p* < 0.05.

			2015–2019	2020	% Change	*t*-Test
**1 March–30 April**	**CO**	Median	0.46	0.20	−56	
		Mean	0.51	0.26	−48	15.36
		St. Dev.	0.25	0.09		
	**NO_2_**	Median	51.4	26.8	−48	
		Mean	53.4	32.4	−39	12.04
		St. Dev.	14.2	18.3		
**10 March–30 April**	**CO**	Median	0.45	0.19	−58	
		Mean	0.50	0.25	−51	15.63
		St. Dev.	0.08	0.09		
	**NO_2_**	Median	51.4	24.5	−52	
		Mean	53.3	26.8	−50	15.72
		St. Dev.	5.2	9.9		
**22 March–30 April**	**CO**	Median	0.44	0.18	−60	
		Mean	0.49	0.22	−56	16.62
		St. Dev.	0.08	0.06		
	**NO_2_**	Median	50.4	21.8	−57	
		Mean	51.9	25.5	−51	20.99
		St. Dev.	4.9	6.3		

**Table 4:** Average concentrations of PM_10_ at individual monitoring stations. Data are expressed in µg·m^−3^. Parametric *t*-test at *p* < 0.05. The italics indicates not-significant *t*-test.

		2015–2019				2020				% Change				*t*-Test			
		IND	GC	CS	DB	IND	GC	CS	DB	IND	GC	CS	DB	IND	GC	CS	DB
**PM_10_**	01/01–/02/29	29	32	31	35	31	32	36	31	6	1	19	−13	−*1.3*	−*0.2*	−3.3	3.4
	03/01–04/30	33	34	34	39	19	19	24	18	−44	−43	−30	−53	10.3	12.9	6.2	16.0
	05/01–07/31	27	29	27	33	17	17	18	20	−35	−42	−34	−38	11.7	13.8	9.5	14.7

**Table 5:** Changes in CO (mg·m^−3^), NO_2_ (µg·m^−3^), and PM_10_ (µg·m^−3^) concentrations, assessed in short periods (6 days) from the start of the lockdown.

			2015–2019	2020	% Change	*t*-Test
11 March–16 March	**CO**	Median	0.46	0.32	−31	5.054
		Mean	0.50	0.37	−27	
	**NO_2_**	Median	54	38	−30	6.648
		Mean	56	43	−23	
	**PM_10_**	Median	29.1	22.1	−24	2.99
		Mean	29.4	22.3	−24	
17 March–22 March	**CO**	Median	0.52	0.22	−58	11.5
		Mean	0.58	0.30	−49	
	**NO_2_**	Median	57	28	−52	17.462
		Mean	59	31	−48	
	**PM_10_**	Median	27.1	18.6	−31	5.014
		Mean	33.6	19.1	−43	
23 March–28 March	**CO**	Median	0.48	0.16	−66	15.86
		Mean	0.50	0.20	−61	
	**NO_2_**	Median	53	25	−52	15
		Mean	54	29	−47	
	**PM_10_**	Median	34.6	14.1	−59	7.49
		Mean	33.8	13.3	−61	

Results of the parametric *t*-test at *p* < 0.05.

**Table 6:** Statistical description of the data from 1 January to 29 February.

1 January–29 February				
		2015–2019	2020	% Change
**CO**	Median	0.56	0.50	−11
	Mean	0.63	0.66	6
	St. Dev.	0.34	0.50	
**NO_2_**	Median	52	51	−3
	Mean	54	54	0
	St. Dev.	6	11	
**PM_10_**	Median	31	32	3
	Mean	32	33	2
	St. Dev.	6	9	

CO data expressed in mg·m^−3^, NO_2_ data expressed in µg·m^−3^, PM_10_ data expressed in µg·m^−3^.
